# Systematic quantification of the dynamics of newly synthesized proteins unveiling their degradation pathways in human cells†

**DOI:** 10.1039/c9sc06479f

**Published:** 2020-03-10

**Authors:** Ming Tong, Johanna M. Smeekens, Haopeng Xiao, Ronghu Wu

**Affiliations:** School of Chemistry and Biochemistry and the Petit Institute for Bioengineering and Bioscience, Georgia Institute of Technology Atlanta Georgia 30332 USA ronghu.wu@chemistry.gatech.edu +1-404-894-7452 +1-404-385-1515

## Abstract

Proteins are continuously synthesized during cell growth and proliferation. At the same time, excessive and misfolded proteins have to be degraded, otherwise they are a burden to cells. Protein degradation is essential to maintain proteostasis in cells, and dysfunction of protein degradation systems results in numerous diseases such as cancer and neurodegenerative diseases. Despite the importance of protein degradation, the degradation pathways of many proteins remain to be explored. Here, we comprehensively investigated the degradation of newly synthesized proteins in human cells by integrating metabolic labeling, click chemistry, and multiplexed proteomics, and systematic and quantitative analysis of newly synthesized proteins first revealed the degradation pathways of many proteins. Bioinformatic analysis demonstrates that proteins degraded through two major pathways have distinct properties and functions. Proteins degraded through the ubiquitin-proteasome pathway contain more disordered structures, whereas those through the autophagy-lysosome pathway have significantly higher hydrophobicity. Systematic and quantitative investigation of the dynamics of newly synthesized proteins provides unprecedented and valuable information about protein degradation, which leads to a better understanding of protein properties and cellular activities.

## Abstract 2

Systematic quantification of the dynamics of newly synthesized proteins first reveals the degradation pathways of many proteins in human cells, and proteins degraded through each of the two major pathways have distinct properties and functions.

## Graphical abstract

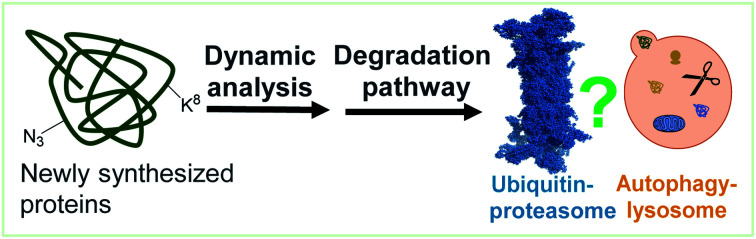

## Introduction

The maintenance of proteostasis is vital for cell survival, growth, and division.^1,2^ Protein degradation is responsible for the removal of unnecessary proteins, such as misfolded and excessive proteins, and is essential to maintain protein homeostasis. In mammalian cells, there are two major types of protein degradation pathways, *i.e.* the ubiquitin-proteasome and the autophagy-lysosome pathways. In the ubiquitin-proteasome pathway, proteins are ubiquitinated by ubiquitin ligase complexes and then degraded by the proteasome,^3^ while in the autophagy-lysosomal pathway, proteins and organelles are captured in the autophagosome and further degraded by proteases in the lysosome.^4^ Dysfunctional protein degradation pathways result in numerous diseases, including cancer, neurodegenerative disorders, and cardiovascular diseases.^5,6^ Systematic investigation of protein degradation and dynamics will help us gain a better understanding of protein degradation pathways, and identify the underlying mechanisms of protein degradation.

There have been many reports studying the degradation of individual proteins.^7,8^ However, most of the existing methods rely on antibodies and fluorescence probes to investigate individual proteins, which limit their capacities for large-scale analysis of protein degradation. In recent years, mass spectrometry (MS)-based proteomics has provided an opportunity to comprehensively characterize proteins,^9–21^ including protein dynamics.^22–25^ For instance, Doherty *et al.* studied the stability of nearly 600 proteins in human A549 cells by using a dynamic stable isotope labeling by amino acids in cell culture (SILAC) approach.^22^ Mathieson *et al.* determined the half-lives of several thousand proteins in human cells using a SILAC-based method and found the architecture-dependent turnover of complex subunits.^26^ Martin-Perez *et al.* also employed a SILAC-based method to measure the turnover rates of over 3000 yeast proteins and found that actively used proteins had a faster turnover rate.^27^ However, some potential issues could exist in the SILAC-based methods. First, amino acid recycling exists, which impacts the quantification of protein dynamics, including protein degradation. Second, proteins are continuously synthesized during cell growth, and analysis of protein degradation could be complex. Furthermore, low-abundance proteins or proteins with high degradation rates may not be analyzed.

Both protein synthesis and degradation are extremely important to maintain proteostasis, and they need to be well-balanced in cells.^1,25^ Newly synthesized proteins are those synthesized within a relatively short period of time. Compared with existing proteins, newly synthesized ones may be more dynamic because a portion of them being misfolded and an excessive amount of newly synthesized proteins need to be promptly degraded.^28,29^ The regulation of newly synthesized proteins is especially important to maintain protein homeostasis in cells, including the degradation of unfolded or improperly folded proteins. In cells, there are many high-abundance proteins that mask signals from low-abundance proteins during MS analysis, and thus effective separation and enrichment of those newly synthesized proteins, many of which have low abundance, are critical for comprehensive analysis of their degradation by MS. In addition, they need to be distinguished from existing proteins. With the development of metabolic labeling^30,31^ and bioorthogonal chemistry,^32,33^ it is possible to selectively tag and separate newly synthesized proteins for further analysis.

Here we integrated metabolic labeling, click chemistry and multiplexed proteomics to comprehensively investigate the dynamics of newly synthesized proteins and their degradation pathways. This method allowed us to selectively enrich newly synthesized proteins and distinguish them from existing ones. Parallel experiments were performed to study protein degradation in MCF-7 cells and measure their half-lives with or without the inhibition of each major degradation pathway: bortezomib (Btz) for the proteasome pathway and 3-methyladenine (3-MA) for the lysosome pathway. Around 3000 newly synthesized proteins were quantified in each experiment, and a total of 4042 proteins were quantified and their half-lives spanned a wide range from a few minutes to over 200 hours. Systematic and quantitative analysis of the dynamics of newly synthesized proteins first revealed that 868 proteins were degraded through the ubiquitin-proteasome pathway while 228 were through the autophagy-lysosome pathway. Bioinformatic analysis demonstrates that proteins degraded through two major pathways have distinct properties and functions. This research provides unprecedented and valuable information regarding protein dynamics, protein degradation pathways and protein properties correlated with their degradation.

## Results

### Identification and quantification of newly synthesized proteins

The combination of metabolic labeling and bioorthogonal chemistry has been proven to be very powerful to label and study proteins.^30,34–40^ Newly synthesized proteins can be labeled with an noncanonical amino acid, such as azidohomoalanine (AHA) that is a methionine analog with an azido group.^30^ The functional group on the labeled proteins serves as a chemical handle for their selective enrichment. Here we integrated metabolic labeling, click chemistry and MS-based proteomics to systematically study the dynamics of newly synthesized proteins and their degradation pathways.

**Fig. 1:**
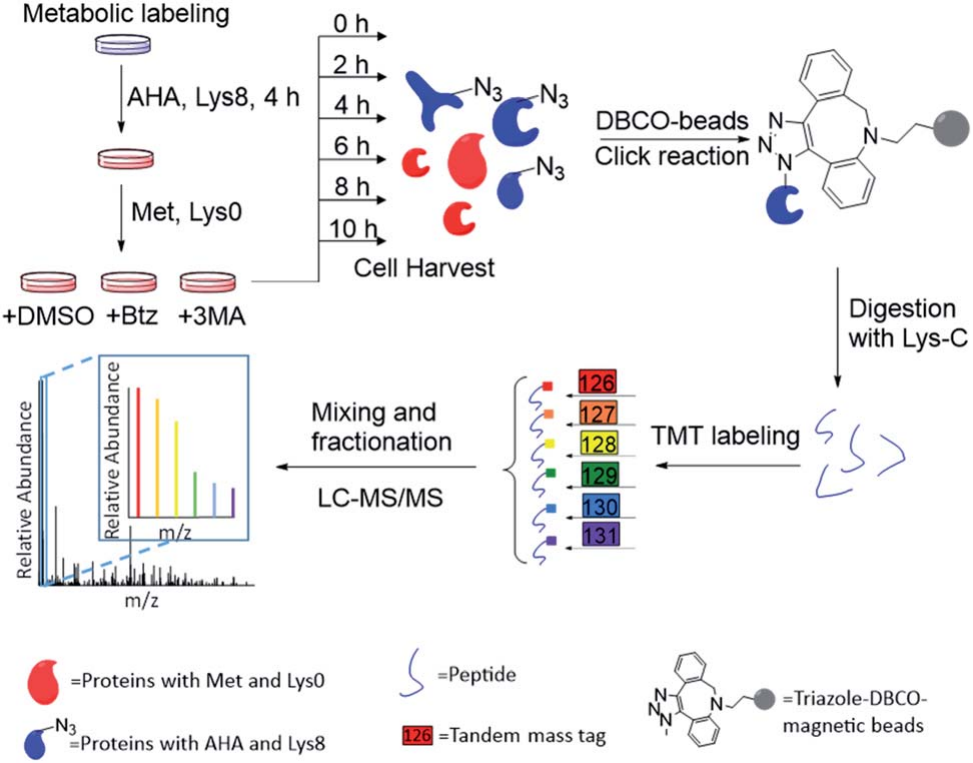
Experimental procedure for quantifying the degradation of newly synthesized proteins and measuring their half-lives in parallel experiments.

The experimental procedure is shown in Fig. 1, and a detailed description is provided in ESI.† Briefly, around 3 × 10^7^ MCF-7 cells were equally passaged to six flasks. After 24 hours, cells were cultured in the medium containing heavy lysine (Lys^8^, ^13^C6 and ^15^N2) and AHA for 4 hours. After metabolic labeling, we switched the media to normal Dulbecco's Modified Eagle Medium (DMEM) with Lys^0^ and methionine. Simultaneously, either Btz, 3-MA or dimethyl sulfoxide (DMSO, as a control) was added to the culture medium, respectively, for each experiment. The numbers of cells were kept as similar as possible before switching the media. Then cells were harvested at 0, 2, 4, 6, 8, and 10 h, respectively, after the media switch. Newly synthesized proteins labeled with AHA were selectively enriched through the copper-free click reaction with dibenzocyclooctyne (DBCO)-derivatized beads; heavy lysine labeling allowed us to unambiguously distinguish them from existing proteins. The beads were stringently washed to remove non-specific binding proteins. Enriched proteins were reduced with 5 mM dithiothreitol (DTT) and alkylated with 15 mM iodoacetamide. After on-bead digestion with Lys-C, peptides from the samples at different time points were each chemically labeled with the sixplex tandem mass tag (TMT) reagents. The reaction was quenched by adding hydroxylamine. Samples were mixed, purified and then separated into 20 fractions by high-pH reversed-phase HPLC. Each fraction was further purified, and then analyzed by LC-MS/MS.

**Fig. 2:**
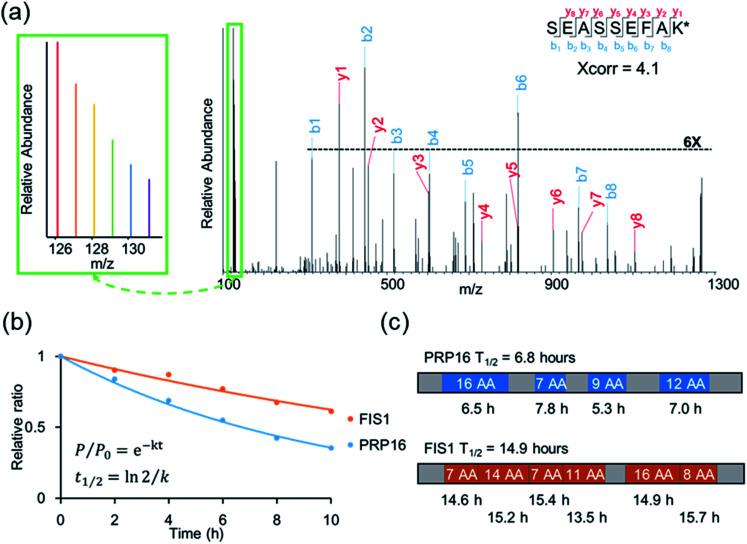
(a) Example tandem mass spectrum of SEASSEFAK* (* refers to heavy lysine), and the intensities of the reporter ions enabled us to accurately quantify the relative abundance change as a function of time (a larger view is in the left box). (b) Examples of the curve fitting and computation for protein half-lives. (c) The half-life of each quantified peptide in two example proteins and the half-life of the corresponding protein.

An example of peptide identification and quantification is displayed in Fig. 2(a). The peptide SEASSEFAK* (* refers to heavy lysine) was confidently identified with an Xcorr of 4.1. This peptide is from PRP16, a pre-mRNA-splicing factor ATP-dependent RNA helicase, which may be involved in pre-mRNA splicing and mRNA exporting from the nucleus. The intensities of the reporter ions enabled us to accurately quantify the relative abundance change as a function of time.

Protein half-lives were calculated by fitting six ratios (the ratio is set to 1 for the first time point) to an exponential decay equation, as reported previously (Fig. 2(b)).^25^ The median intensity of the reporter ions were calculated from four unique peptides for PRP16, and the ratio at each of the six time points was 1, 0.84, 0.69, 0.55, 0.42, or 0.35, respectively. Those ratios were then fitted to the following equation, *F*(*t*) = e^−0.101*t*^, and the half-life was calculated to be 6.8 (ln(2)/0.101) hours. The half-life of FIS1 was determined by the same process. Overall, eleven total and six unique peptides from this protein were quantified, and its half-life was 14.9 hours. In addition, as an example, the half-life of each unique peptide was also calculated and compared with the parent proteins (Fig. 2(c)). The majority of peptides are within ±10% of the half-life of the parent protein, which shows the relatively high reproducibility of this method.

### Comprehensive analysis of protein half-lives in MCF-7 cells

In untreated MCF-7 cells, we quantified the dynamics of 2970 newly synthesized proteins, and calculated their half-lives (Table S1†). When proteins are very stable, the half-lives calculated from the curve fitting may not be accurate. In this case, for a small group of very stable proteins (*k* < 0.0034 h^−1^), their half-lives were set to 200 hours. Protein half-lives span a wide range from 25 minutes to over 200 hours. The half-life distribution is shown in Fig. S1† and the median half-life was 9.8 hours. This value is in reasonably good agreement with that measured using an MS-independent method, *i.e.* a bleach-chase experiment with fluorescence detection in human cancer cells.^41^ In that work, 100 proteins were measured and the median half-life was 8.2 hours. Here, the median half-life of nearly 3000 quantified newly synthesized proteins is 9.8 hours, which is much shorter than the doubling time of the cells (∼24 hours). Compared with existing proteins, newly synthesized ones are typically more dynamic with shorter half-lives. The half-lives of over 91% quantified proteins are from 3 to 20 hours, while 127 proteins have short half-lives of <3 hours and 116 proteins have long half-lives of >20 hours.

Gene Ontology analysis with Database for Annotation, Visualization and Integrated Discovery (DAVID)^42^ was performed to determine the cellular localization of those proteins with short (<3 hours) or long (>20 hours) half-lives. As shown in Fig. S2,† among short-lived proteins, those related to the nuclear lumen, proteasome and ribosomal subunits were highly enriched. For instance, the half-lives of RPL15 (60S ribosomal protein L15), PSMD6 (26S proteasome non-ATPase regulatory subunit 6) and JAK1 (tyrosine-protein kinase JAK1) were 1.2, 1.8 and 1.8 hours, respectively. It has been reported that ribosomal proteins, especially those located in the nucleolus, have fast turnover rates.^23^ JAK1 was reported to be a highly unstable protein, and the current result corresponds well with the half-life of 1.5 h in previous research.^43^ On the contrary, among long-lived proteins, those located in the extracellular exosome, endomembrane system and mitochondrion were over-represented (Fig. S2(b)†). Moreover, the half-lives of histone proteins are relatively long: 23.9 h for HIST2H2BC, 24.1 h for HIST1H1B and 20.0 h for HIST1H1D.

**Fig. 3:**
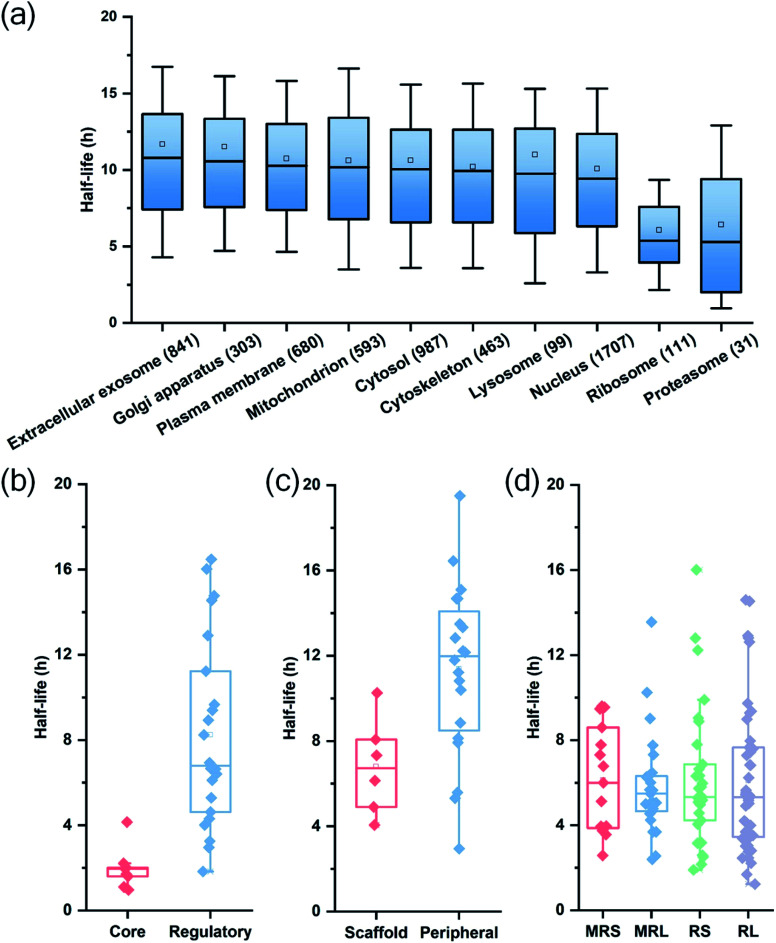
Half-life distributions of newly synthesized proteins in (a) different cellular compartments and protein complexes, (b) the core and regulatory subunits of the proteasome complex, (c) the scaffold and peripheral subunits in the nuclear pore complex, (d) the mitochondrial (mitochondrial ribosomal small subunit (MRS) and mitochondrial ribosomal large subunit (MRL)) and cytosolic (cytosolic ribosomal small subunit (RS) and cytosolic ribosomal large subunit (RL)) ribosome complexes.

Among quantified proteins, those in the extracellular exosome had the longest median half-life. Proteins in the mitochondrion, the cytosol and the nucleus had a median half-life close to the overall median of 9.8 h (Table S2† and Fig. 3(a)). The half-lives of proteins in the proteasome and ribosome complexes were relatively shorter and their median half-lives were 6.8 and 6.3 h, respectively. This trend is in good agreement with previous studies in both mammalian and yeast cells.^26,27^

Protein half-lives in different protein complexes and their subunits were also assessed and compared (Table S3† and Fig. 3(b)–(d)). Here we looked into three protein complexes, *i.e.* the proteasome, the ribosome, and the nuclear pore complex (NPC). Differences between different subunits of the complexes were observed. For instance, the half-lives of proteins in the 20S core subunit were dramatically shorter (*p* < 0.001) than those of proteins in the 19S regulatory subunit of the proteasome (Fig. 3(b)). Protein half-lives in the peripheral structure of the NPC were longer (*p* < 0.05) than those in the scaffold part (Fig. 3(c)), which differs from a previous study.^26^ We reason that proteins in the complexes are typically synthesized in excessive amounts and degraded continuously.^28,44^ According to a previous study about the dynamic organization of NPC, the residence periods of the peripheral proteins Nup153 and Nup50 were 20 s and 1 min, respectively, while the residence periods of the scaffold proteins Nup133 and Nup205 were more than 35 h.^45^ Therefore, newly synthesized peripheral proteins Nup153 and Nup50 may be more rapidly assembled to NPC, contributing to their longer half-lives (16.4 and 19.5 h).

For the ribosome complexes, the median half-life of proteins in the mitochondrial ribosome was slightly longer compared to proteins in the cytosolic ribosome, and the median half-life of proteins from the small subunits was longer than that from the large subunits (Fig. 3(d)).

### Quantification of protein dynamics with the inhibition of degradation pathways

The proteasomal machinery is responsible for protein degradation, and multiple inhibitors for the proteasome have been reported.^46,47^ Bortezomib is most commonly used as a potent inhibitor of the proteasome. The crystal structure of the proteasome–Btz complex revealed that the boronic acid moiety of Btz specifically binds to the hydroxyl group of threonine in the active site. This interaction is stabilized by hydrogen bonds between the nearby groups in the proteasome and the Btz peptide backbones,^47^ which results in the specific and stable proteasome inhibition.

**Fig. 4:**
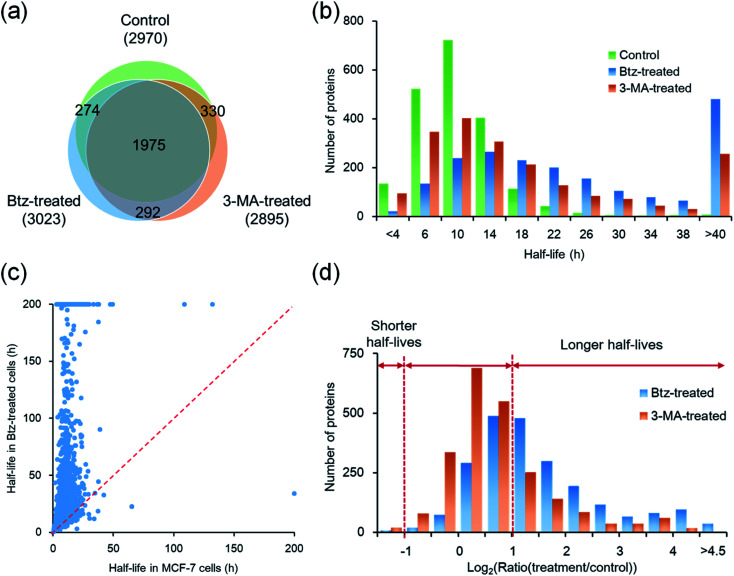
Comparison of the half-lives of newly synthesized proteins between untreated, Btz-treated and 3-MA-treated MCF-7 cells. (a) Overlap of proteins quantified in three parallel experiments. (b) Distributions of protein half-lives in MCF-7 cells with or without the inhibition of the proteasome or lysosome pathway. (c) Comparison of protein half-lives between untreated and Btz-treated MCF-7 cells. (d) Distributions of protein half-life ratios in Btz-treated or 3-MA treated *vs.* untreated MCF-7 cells.

After cells were treated with Btz, a total of 3023 proteins were quantified and their half-lives were calculated, which are listed in Table S1† (Fig. 4(a)). It is expected that many proteins have longer half-lives with the Btz treatment, especially for those degraded through the proteasome. The median half-life for quantified proteins was 21.7 h, which is more than twice as long as untreated cells (9.8 h); the half-life distributions of overlapped proteins (1975) in three parallel experiments are displayed in Fig. 4(b). As shown in Fig. 4(a), 2249 proteins were quantified in cells with or without the Btz treatment. The half-lives of >60% proteins increased more than two-fold while the half-lives of very few proteins (only 9) decreased over 2-fold (Fig. 4(c)).

For instance, ARPC5 functions as a component of the Arp2/3 complex which is involved in the regulation of actin polymerization, and it was reported to be degraded through the ubiquitin-proteasome pathway.^7^ ARPC5 had a half-life of 3.7 h in untreated cells while its half-life increased >3-fold (11.9 h) in proteasome-inhibited cells. CDK5RAP3 is a tumor suppressor which controls cell proliferation and inhibits the NF-kappa-B-mediated gene transcription.^8^ In MCF-7 cells, CDK5RAP3 had a half-life of 10.8 h, which increased to 54.7 h after the Btz treatment.

In order to further understand protein degradation, we also quantified protein dynamics after the inhibition of the other major protein degradation pathway, *i.e.* the autophagy-lysosome pathway. 3-Methyladenine was often reported to effectively inhibit the autophagy-lysosome pathway.^48,49^ In this experiment, 2895 proteins were quantified and their half-lives were obtained for 3-MA-treated cells (Fig. 4(a)). The median half-life of these quantified proteins is 13.4 h, which is 3.6 h longer than that from untreated cells.

Among 2305 proteins quantified in cells with or without the treatment of 3-MA, the half-lives of 630 proteins increased more than 2-fold (Fig. 4(d) and S3†). For example, RAB5A, a small GTPase Rab protein, is a key regulator of intracellular membrane trafficking and autophagosome assembly. VAMP7 is required in the transport of proteins from early endosome to the lysosome. Both proteins are known to be degraded through the lysosome after the fusion between the autophagosome and the lysosome. Their half-lives increased 6.3- and 7.5-fold after the 3-MA treatment, indicating the effective inhibition of the autophagy-lysosome pathway.

Taken together, a total of 4042 proteins were quantified across three parallel experiments (Table S4†); as shown in Fig. 4(a), 1975 proteins (∼65% in each experiment) were quantified in all three experiments. The half-life distributions of overlapped proteins in untreated, Btz-treated and 3-MA-treated cells are displayed in Fig. 4(b). With the treatment of Btz, the distribution shifted dramatically towards longer half-lives.

### Comparison of proteins quantified with the inhibition of the proteasome or lysosome pathway

Although both the ubiquitin-proteasome and autophagy-lysosome pathways are the major protein degradation pathways, it is believed that more proteins are degraded through the proteasome. This is also supported by the median half-life differences in Btz-treated (21.7 h), 3-MA-treated (13.4 h) and untreated cells (9.8 h). In the current experiments, the half-lives of 1368 newly synthesized proteins were up-regulated by >2-fold in Btz-treated cells while less than half of the newly synthesized proteins (630) were up-regulated by >2-fold in 3-MA-treated cells (Table S4†).

**Fig. 5:**
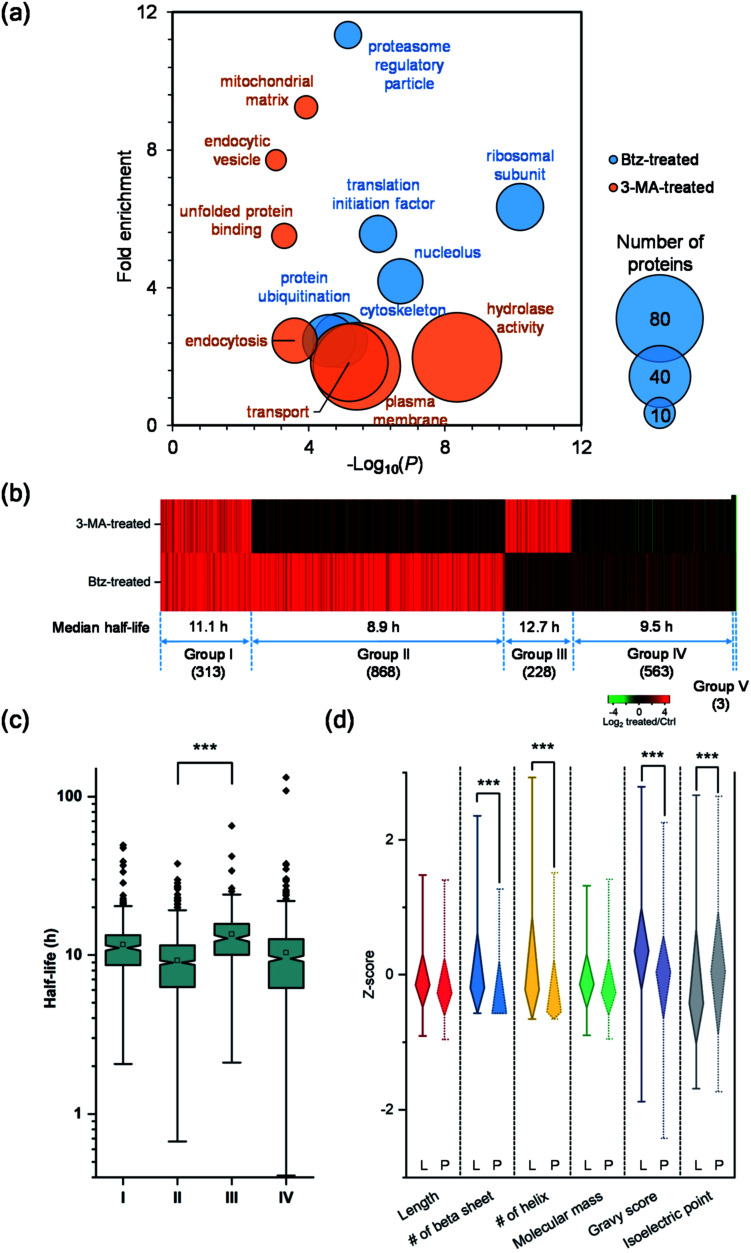
Comparison of the half-lives of proteins in untreated, Btz-treated and 3-MA-treated MCF-7 cells, and evaluation of their degradation pathways. (a) Clustering of proteins with two-fold longer half-lives after Btz or 3-MA treatment based on cellular component, molecular function and biological process. The size of each circle represents the number of proteins in each cluster. (b) Heatmap comparing the half-life changes of newly synthesized proteins after the 3-MA (top) or Btz (bottom) treatment. Each line represents a single protein. Proteins were clustered and combined into five groups based on the half-life changes. (c) Differences in the distribution of protein half-lives within different groups were assessed by unpaired *t* test (significance levels are represented as *** (*P* < 0.001), ** (*P* < 0.01) and * (*P* < 0.05)). (d) Comparison of the properties (*Z*-score transformed values of six physiochemical properties) of proteins degraded through either the proteasome pathway (Group II, marked as “P”) or the lysosome pathway (Group III, marked as “L”).

The up-regulated proteins were further analyzed using DAVID and Protein Analysis Through Evolutionary Relationships (PANTHER);^50^ selective results are included in Fig. 5(a). For Btz-treated cells, proteins located in the proteasome regulatory particle, ribosomal subunit, cytoskeleton and nucleolus were highly enriched. Proteins related to translational initiation and ubiquitination were also overrepresented. For 3-MA-treated cells, the clustering results were dramatically different from Btz-treated cells. Proteins located in the plasma membrane, endocytic vesicle and mitochondrial matrix were highly enriched. Proteins related to transport, unfolded protein binding and hydrolase activity were enriched as well.

To determine which pathway is responsible for the degradation of each protein, we divided all proteins into five groups (Fig. 5(b)). Group I contains 313 proteins, whose half-lives increased at least two-fold due to treatment with Btz or 3-MA. The inhibition of either the ubiquitin-proteasome or autophagy-lysosome pathway dramatically affected (log_2_ ratio > 1) the degradation of those proteins. For instance, RPL19 is a component of the ribosomal large subunit, and its half-life increased 3.3- and 2.1-fold after Btz or 3-MA treatment, respectively. Group II has 868 proteins, and their half-lives increased more than two-fold after Btz treatment, but not 3-MA treatment, indicating that those proteins were degraded only through the ubiquitin-proteasome pathway. On the contrary, Group III contains 228 proteins which were degraded only through the autophagy-lysosome pathway. The median half-life in untreated cells were 8.9 h for Group II and 12.7 h for Group III, and the difference in half-life distributions between Group II and III is significant with a *P* value < 0.0001 (Fig. 5(c)). This is consistent with the common belief that short-lived proteins are typically degraded through the ubiquitin-proteasome pathway while proteins degraded through the autophagy-lysosome pathway are relatively longer-lived.^51,52^

Five-hundred and sixty-three proteins in Group IV were defined as uncertain because their half-lives stayed relatively consistent (−1 < log_2_ ratio < 1) with the treatment of Btz or 3-MA, compared with those in untreated cells. They might be relatively more stable during the inhibition of either major pathway, and/or these proteins may be degraded through other pathways. Surprisingly, three proteins, *i.e.* histone H4, lanosterol 14-alpha demethylase and POTE ankyrin domain family member E, were found to have dramatically shorter (log_2_ ratio < −1) half-lives after the Btz or 3-MA treatment. More studies need to be performed to understand this interesting phenomenon.

To better understand protein degradation, we performed further analysis on the physiochemical properties between proteins degraded only through the ubiquitin-proteasome or autophagy-lysosome pathway. Selected results are displayed in Fig. 5(d) and S4.† For instance, hydrophobic amino acid residues more frequently existed in proteins degraded through the lysosome pathway, including the percentages of valine, isoleucine and phenylalanine being significantly higher. However, basic amino acid residues were more frequently found in proteins degraded through the proteasome pathway, especially lysine (Fig. S4†). This can be explained by the fact that ubiquitination typically occurs on the lysine residue before protein degradation by the proteasome. As shown in Fig. 5(d), protein length and molecular weight did not have notable differences between proteins degraded through either pathway. However, proteins degraded through the proteasome pathway contained more disordered structures, *i.e.* the lower numbers of beta sheets and helix. Consistent with the frequencies of amino acid residues, the hydrophobicities (GRAVY score) of proteins were significantly higher in proteins degraded through the lysosome pathway. Interestingly, higher isoelectric points were found in proteins degraded through the proteasome pathway, which matches very well with the higher frequencies of basic amino acid residues.

### Degradation pathways of proteins in complexes

**Fig. 6:**
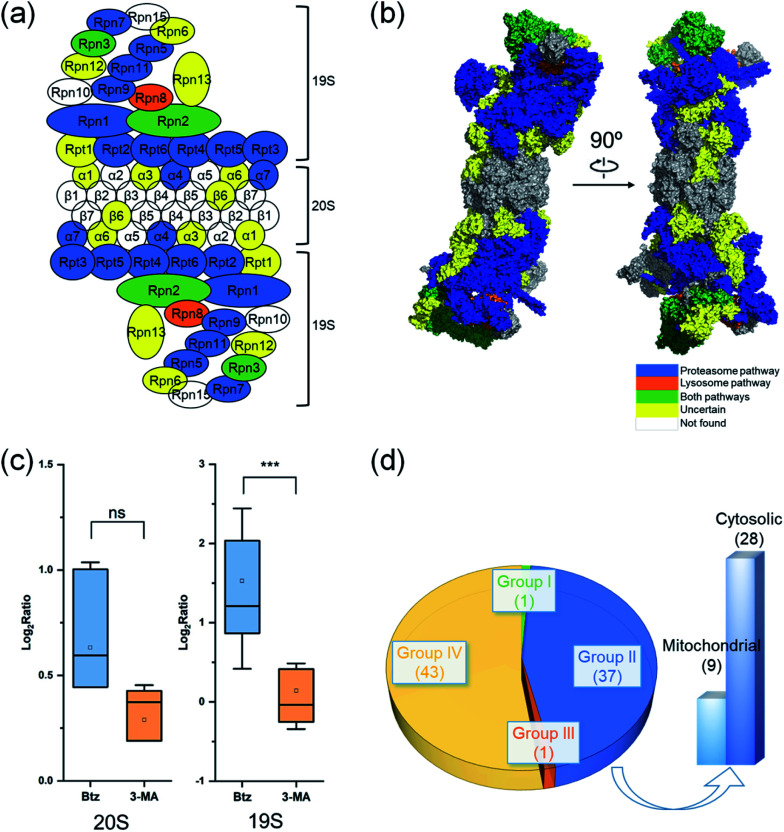
Degradation of proteins from the proteasome, cytosolic ribosome and mitochondrial ribosome complexes. The degradation pathway of each protein is shown in (a) the scheme of the proteasome and (b) the structure of the proteasome. Each protein is colored based on its degradation pathway. Proteins degraded through the ubiquitin-proteasome pathway are in blue, through the autophagy-lysosome pathway are in orange, through both pathways are in green, proteins with an uncertain degradation pathway are in yellow, and unquantified proteins are in white. (c) Distribution of log_2_ ratios of the protein half-lives from the 19S regulatory subunit and the 20S core subunit in the treated and untreated experiments. (d) Distribution of ribosomal protein degradation pathways.

Proteins in a complex work together to fulfill the function of the complex, and their degradation is of particular interest. Here we studied proteins in the proteasome and the ribosome (cytosolic and mitochondrial) based on their structures in the literature.^53–55^ Proteins are displayed in different colors based on their degradation pathways: proteins degraded through the proteasome are in blue, through the autophagy-lysosome pathway are in orange, through both pathways are in green and through an uncertain pathway are in yellow (Fig. 6 and S5†).

In untreated MCF-7 cells, proteins in the 20S core subunit had much shorter half-lives compared with those in the 19S regulatory subunit. As shown in Fig. 6(a) and (b), different degradation pathways were found for proteins in the 20S and 19S subunits (Table S5†). Most proteins in the 19S regulatory subunit were degraded through the proteasome, except Rpt1, Rpn6, Rpn12 and Rpn13, whose ratios are higher than 1.0 (1.5, 1.1, 1.8 and 1.3, respectively) with the inhibition of the proteasome. This suggests a high likelihood that they were also degraded through the proteasome pathway. The half-life of Rpn8 was 6.9 h in untreated cells and increased to 12.5 and 15.3 h under the Btz or 3-MA treatment, respectively. This protein was likely degraded through both pathways. On the contrary, only two proteins in the 20S core subunit were degraded by the proteasome. The responses of 19S regulatory subunit proteins to the Btz or 3-MA treatment were dramatically different (*P*-value < 0.001), while no significant difference was found for 20S core subunit proteins (Fig. 6(c)).

The cytosolic and mitochondrial ribosomes are responsible for protein synthesis in cells. Overall, we quantified 82 ribosomal proteins from the 60S and 40S ribosome subunits and the 28S and 39S mitochondrial ribosome subunits. Thirty-seven proteins were degraded through the proteasome when using >2-fold increase of the half-life as the threshold (Fig. 6(d) and S5†).

However, the half-lives of nearly all ribosomal proteins increased with the inhibition of the proteasome. For example, the half-lives of MRPL37 and RPL27 increased 2 fold, and proteins RPS25, MRPS2, RPL10, RPS9, RPL29, MRPL41, RPS18 and RPL13A had a 1.9 fold increase in their half-lives with the inhibition of the proteasome, but their half-lives remained similar after the 3-MA treatment (Table S5(b)†). These proteins very likely were also degraded through the proteasome pathway. The current results indicate that ribosomal proteins were mainly degraded through the proteasome.

## Discussion

Protein synthesis and degradation are equally important for protein homeostasis in cells and they need to be well-balanced. Newly synthesized proteins are typically more dynamic because a portion of them cannot be folded properly and excess amounts of them are often synthesized, which need to be promptly degraded. Compared to total cellular proteins, investigating the dynamics of newly synthesized proteins is not well studied. In this work, we selectively enriched newly synthesized proteins labeled with an azido group, which allowed us to study their dynamics and investigate proteins with low abundance or high degradation rates.^35^ Overall, nearly 3000 newly synthesized proteins were quantified in MCF-7 cells. Their half-lives spanned a wide range from 25 minutes to over 200 hours with a median half-life of 9.8 hours. This corresponds well with the median half-life (8.2 hours) of 100 proteins in the NCI-H1299 cell line in a bleach-chase experiment with fluorescence detection.^41^

In this work, the protein half-lives were much shorter than those from the SILAC-based proteomic experiments.^23,25,56^ One contributing factor is that newly synthesized proteins are more dynamic than existing proteins. Previous studies illustrated that up to 30% of newly synthesized proteins were defective ribosomal products, which were rapidly degraded after their synthesis.^29^ Furthermore, using SILAC-based methods, amino acid recycling is an inherent issue, which results in longer protein half-lives. By using the current method, AHA and heavy lysine may still be recycled, but the possibility of recycling both AHA and heavy lysine in a single newly synthesized peptide and protein is very low.

Using the TMT method, the quantification of peptides could result in ratio suppression due to potential interferences.^57^ In order to minimize potential interferences, we took several preventative measures. First, each sample was fractionated into twenty fractions using HPLC, which made each fraction much less complex. Second, a long gradient during LC-MS analysis allowed peptides to be further separated. Third, a narrower isolation window was used, *i.e.* 1.2 D instead of the commonly used 2.0 D. As a result, potential interference may not be an issue in this work, especially considering the enriched samples (newly synthesized proteins) are less complex compared to the whole proteome. If the ratio suppression is a serious problem, the half-lives of quantified proteins would be much longer. However, the protein half-lives are obviously shorter than those obtained using SILAC-based methods,^25,58^ and they correspond well with those measured from an MS-independent method.^41^

Here, we studied protein degradation through investigating the dynamics of newly synthesized proteins, and the half-lives of newly synthesized proteins were calculated from six time points. Proteins at the six time points were quantified simultaneously using the multiplexed proteomics method, which further increased the quantification accuracy. Compared with SILAC methods typically based on two or three time points, the current results are more reliable. In this work, MCF-7 cells were used to study protein degradation, and cell-specific degradation of some proteins may not be excluded, which needs to be further studied.

After the inhibition of the ubiquitin-proteasome or autophagy-lysosome pathway through the treatment of Btz or 3-MA, respectively, the median half-life of newly synthesized proteins increased to 21.7 or 13.4 hours from 9.8 hours in cells without any treatment. Comprehensive analysis of the degradation of newly synthesized proteins with and without the inhibition of either major protein degradation pathway revealed that 868 proteins were degraded through the proteasome while 228 proteins were through the autophagy-lysosome pathway. In addition, 313 proteins were degraded through both pathways. Interestingly, some proteins were found to have shorter half-lives after the inhibition of either degradation pathway, which may be due to the cross-talk between the ubiquitin-proteasome and autophagy-lysosome pathways. Although the mechanisms of the two pathways are dramatically different, several studies reported that there are some correlations between these two major degradation pathways.^59,60^ The inhibition of the proteasome may result in the induction of the unfolded protein response and lead to the activation of the transcriptional factors, ATF4 and IRE1, increasing the activity of the autophagy-lysosome pathway.^61^ In the current work, we identified 41 proteins with longer half-lives after the inhibition of the autophagy-lysosome pathway, but their half-lives decreased with the Btz treatment, including NRAS and 7 Ras-related proteins, which could be due to cross-talk between the two pathways. These proteins may serve as examples to further study the mechanisms of the cross-talk between the ubiquitin-proteasome and autophagy-lysosome pathways.

## Experimental

### Cell culture, metabolic labeling and Btz or 3-MA treatment

Human breast cancer cells (MCF-7, from American Type Culture Collection) were cultured in DMEM supplemented with 1000 mg L^−1^ glucose and 10% FBS. Twenty-four hours before metabolic labeling, cells were equally passaged to six flasks. After 24 hours, cells were grown in depleted DMEM without lysine and methionine for 1 hour and subsequently incubated in DMEM with 10% diFBS, Lys^8^ and AHA for 4 hours. After metabolic labeling, media were replaced with regular DMEM with Lys^0^ and Met. A 1.5 mM Btz stock solution was prepared in DMSO and added to six flasks of cells to a final concentration of 100 nM. A 10 mg mL^−1^ 3-MA stock solution in DMSO was prepared and added to another six flasks of cells to a final concentration of 5 mM. A similar volume of DMSO was added to the flasks of untreated cells that served as a control. For each series of experiments (untreated, Btz-treated and 3-MA treated), one flask of cells was harvested at 0, 2, 4, 6, 8, or 10 hours, respectively, after the media switch.

### Cell lysis, newly synthesized protein enrichment and digestion

Cells were pelleted by centrifugation at 500*g* for 3 min, washed with ice-cold PBS twice, and lysed with end-over-end rotation at 4 °C for 1 hour in RIPA buffer containing 100 mM 4-(2-hydroxyethyl)piperazine-1-ethanesulfonic acid (HEPES) pH = 7.9, 150 mM NaCl, 1% sodium deoxycholate (SDC), benzonase nuclease (10 unit per mL) and complete protease inhibitor cocktail (Roche, 1 pill/10 mL). Lysates were centrifuged at 4696*g* for 10 min and the supernatants were collected. Newly synthesized proteins were enriched through incubating with dibenzocyclooctyne (DBCO)-derivatized beads at 4 °C overnight. The enriched proteins were then reduced by 5 mM DTT (56 °C, 25 min) and alkylated by 15 mM iodoacetamide (RT, 30 minutes in the dark). The beads were washed with 10 volumes of RIPA buffer containing 2.5% sodium dodecyl sulfate (SDS) and 2.5% SDC, 10 volumes of 100 mM HEPES (pH = 8.1) containing 8 M urea, 10 volumes of 50% isopropanol in water, and 10 volumes of 50% acetonitrile (ACN) in water. Proteins bound to the beads were digested with Lys-C at 31 °C overnight in the digestion buffer (50 mM HEPES pH = 8.1, 1.6 M urea and 5% ACN). The digestion was quenched by adding trifluoroacetic acid (TFA) to a final concentration of 0.4%. Supernatant was collected after centrifugation and digested peptides were purified with a 100 mg SepPak tC18 cartridge.

### Peptides labeled with TMT reagents and HPLC fractionation

Purified peptide samples from six time points (0, 2, 4, 6, 8 and 10 hours) were labeled with the sixplex TMT reagents, respectively, according to the manufacturer's protocol. Briefly, peptides were dissolved in 100 μL of 100 mM HEPES buffer, pH = 8.5. Each tube of the TMT reagent was dissolved in 41 μL of anhydrous ACN and transferred into the peptide tube. The reaction lasted for 1 h at room temperature, and then was quenched by adding 10 μL of 5% hydroxylamine. The six labeled peptide samples were combined and desalted. Then the TMT-labeled peptides were fractionated by high-pH reversed-phase high performance liquid chromatography (HPLC) into 20 fractions using a 4.6 × 250 mm 5 μm particle reversed-phase column (Waters) with a 40 min gradient of 5–55% ACN in 10 mM ammonium formate (pH = 10). Each fraction was further purified by the Stage Tip method.^62^

### LC-MS/MS analysis

Each dried peptide sample was suspended in a solvent of 5% ACN and 4% formic acid (FA), and 4 μL was loaded onto a microcapillary column packed with C18 beads (Magic C18AQ, 3 μm, 200 Å, 75 μm × 16 cm) by a Dionex WPS-3000TPLRS autosampler (UltiMate 3000 Thermostatted Pulled Loop Rapid Separation Wellplate Sampler). Peptides were separated by reversed-phase HPLC using an UltiMate 3000 binary pump with an 112-minutes gradient of 3–14%, 4–17%, 8–24%, or 10–28% ACN (0.125% FA), respectively, for each fraction of the TMT labeled samples. The full MS and MS^2^ were detected in a hybrid dual-cell quadrupole linear ion trap – Orbitrap mass spectrometer (LTQ Orbitrap Elite, Thermo Scientific, with Xcalibur 3.0.63 software) using a data-dependent Top15 method.^35^ Each cycle had one full MS scan (resolution: 60 000) in the Orbitrap at the AGC target of 10^6^, followed by up to 15 MS/MS for the most intense ions. The selected ions were excluded from further analysis for 90 s. Ions with a single or unassigned charge were not sequenced. MS/MS scans were activated by HCD at 40.0% normalized collision energy with 1.2 *m*/*z* isolation width. Fragments were detected in the Orbitrap cell with high resolution and high mass accuracy.

### Database searching, data filtering and protein quantification

Raw data files recorded from the mass spectrometer were first converted into the mzXML format. All MS/MS spectra were searched using the SEQUEST algorithm (version 28)^63^ and matched against a database encompassing sequences of all proteins downloaded from the Uniprot human (*Homo sapiens*) plus common contaminants such as keratins. Each protein sequence was listed in both forward and reversed orientations to estimate the false discovery rate (FDR) of peptide and protein identifications. Database searches were performed by using the following parameters: 20 ppm precursor mass tolerance; 0.025 Da product ion mass tolerance; fully digested with Lys-C; up to two missed cleavages; variable modifications: oxidation of methionine (+15.9949) and heavy lysine (+8.0142); fixed modifications: carbamidomethylation of cysteine (+57.0214) and the TMT labeling of lysine and the peptide *N*-terminus (+229.1630).

The target-decoy method was employed to evaluate and control the FDRs of peptide and protein identifications.^64^ Linear discriminant analysis (LDA) was exploited to distinguish correct and incorrect peptide identifications using numerous parameters such as XCorr, ΔCn, and precursor mass error.^65^ After scoring, peptides fewer than seven amino acids in length were discarded and peptide spectral match was filtered to a less than 1% FDR based on the number of decoy sequences in the final data set.

The TMT reporter ion intensities in the MS/MS spectra were used to quantify peptides. The isotopic information provided by Thermo was utilized to calibrate the ion intensities. Only peptides with the reporter ions found at all six channels were further analyzed for quantification. The median intensity at each time point was calculated from all unique peptides for each protein. The degradation rate and the standard deviation were obtained by fitting six ratios (the ratio at the first time point is set as 1) to the exponential decay equation based on the orthogonal distance regression algorithm. The protein half-lives were then computed based on the degradation rates.

## Conclusions

Through the integration of metabolic labeling, click chemistry and MS-based proteomics, we systematically investigated the dynamics of newly synthesized proteins in MCF-7 cells and measured their half-lives. In order to determine the degradation pathways of proteins, we performed the parallel experiments in cells with the inhibition of each major protein degradation pathway. For every experiment, about 3000 newly synthesized proteins were quantified. With the inhibition of either major protein degradation pathway, the median half-life of proteins increased dramatically. The inhibition of the proteasome resulted in a greater than two-fold increase in the median half-life of newly synthesized proteins quantified here. The current results revealed that 868 proteins were degraded through the ubiquitin-proteasome pathway while 228 proteins were degraded through the autophagy-lysosome pathway. In addition, 313 proteins were degraded through both pathways. As expected, the length and molecular weight of proteins did not have notable differences between proteins degraded through either pathway. However, proteins degraded through the ubiquitin-proteasome pathway contained more disordered structures. Consistent with the frequencies of amino acid residues, the hydrophobicities (GRAVY score) of proteins were significantly higher in proteins degraded through the autophagy-lysosome pathway. In addition, notable higher isoelectric points were found in proteins degraded through the ubiquitin-proteasome pathway, which matches very well with the higher frequencies of basic amino acid residues, especially lysine. Lysine is the well-known residue for protein ubiquitination and subsequent degradation in the proteasome. We further analyzed the half-lives of proteins in the proteasome and ribosome complexes, and found that newly synthesized proteins in the regulatory subunit of the proteasome had longer half-lives, most of which were degraded through the proteasome. For the first time, systematic and quantitative investigation of the dynamics of newly synthesized proteins unveils the degradation pathways of many proteins, which facilitates a better understanding of protein properties and cellular activities.

## Data availability

The raw files are available on a publicly accessible website: http://www.peptideatlas.org/PASS/PASS01336. The password is AV8867yw.

## Conflicts of interest

The authors declare no competing interests.

## Supplementary Material

SC-011-C9SC06479F-s001

SC-011-C9SC06479F-s002

SC-011-C9SC06479F-s003

SC-011-C9SC06479F-s004

SC-011-C9SC06479F-s005

SC-011-C9SC06479F-s006
