# Supplementary material for: Systematic quantification of the dynamics of newly synthesized proteins unveiling their degradation pathways in human cells
Source: Chem Sci. 2020 Mar 10;11(13):3557–68. doi: 10.1039/c9sc06479f (PMC8152571; doi:10.1039/c9sc06479f)
Supplement: SC-011-C9SC06479F-s001 [file SC-011-C9SC06479F-s001.pdf]

Electronic Supplementary Material (ESI) for Chemical Science.
This journal is © The Royal Society of Chemistry 2020

SUPPLEMENTARY INFORMATION

Systematic Quantification of the Dynamics of Newly Synthesized Proteins Unveiling Their Degradation Pathways in Human Cells

Ming Tong, Johanna M. Smeekens, Haopeng Xiao, and Ronghu Wu*

School of Chemistry and Biochemistry and the Petit Institute for Bioengineering and Bioscience, Georgia Institute of Technology, Atlanta, Georgia 30332, USA

A list of Supplemental Tables in Excel format:

Table S1: Quantification of the dynamics of proteins and their half-lives in untreated, btz-treated and 3-MA-treated MCF-7 cells

Table S2: Protein half-lives in different cellular compartments

Table S3: Protein half-lives in the proteasome and ribosome complexes

Table S4: Comparison of the half-lives of 4042 quantified proteins in three experiments

Table S5: Degradation pathways of proteins in the proteasome and ribosome complexes

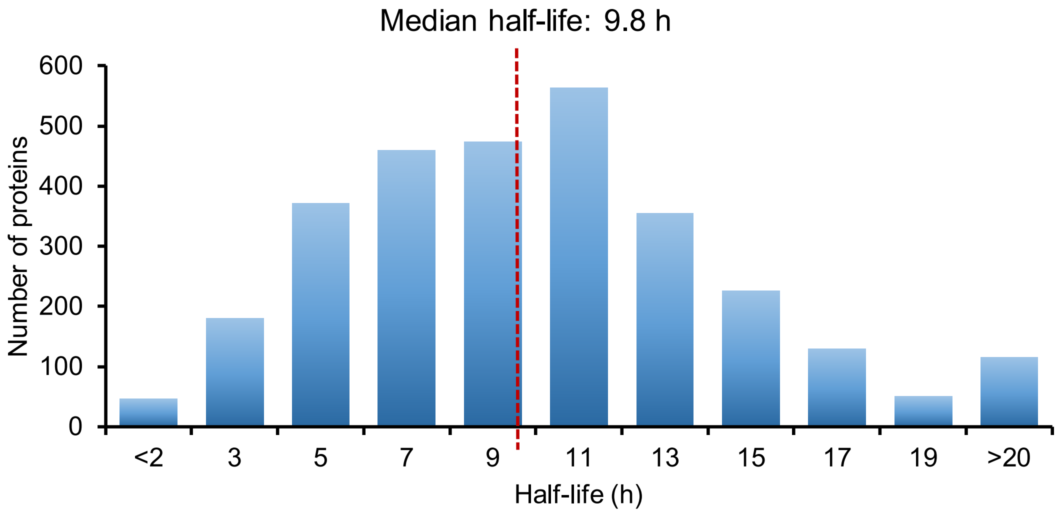

Figure S1. The distribution of the half-lives of newly synthesized proteins in MCF-7 cells.

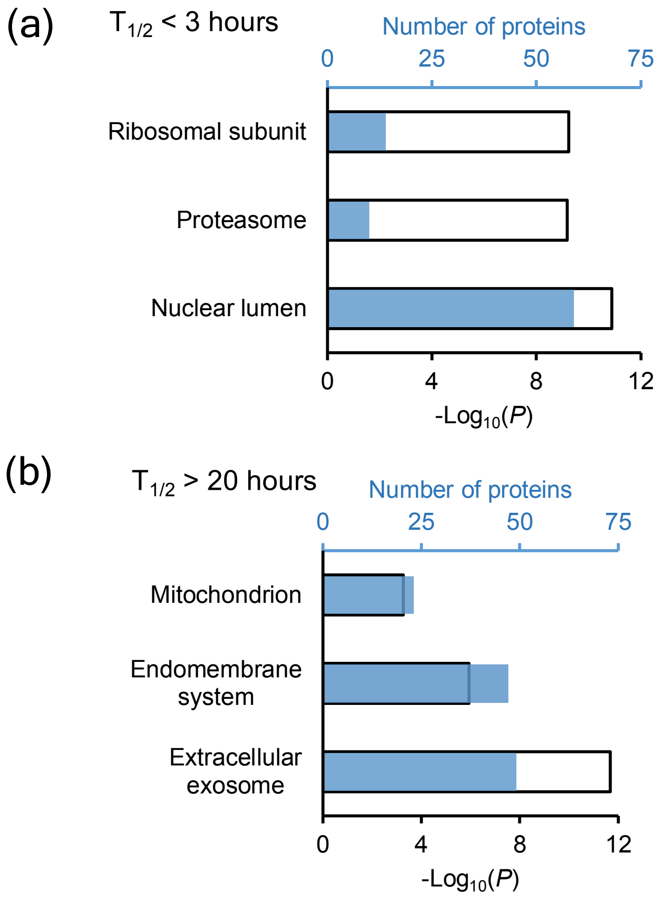

Figure S2. Clustering of proteins with short half-lives (a) and with long half-lives (b) in MCF-7 cells.

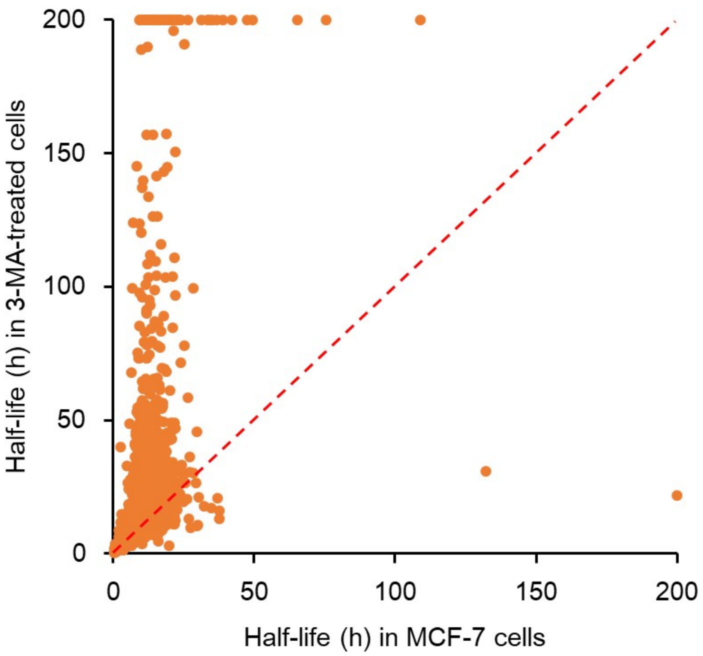

Figure S3. Comparison of protein half-lives quantified in normal and 3-MA-treated MCF-7 cells.

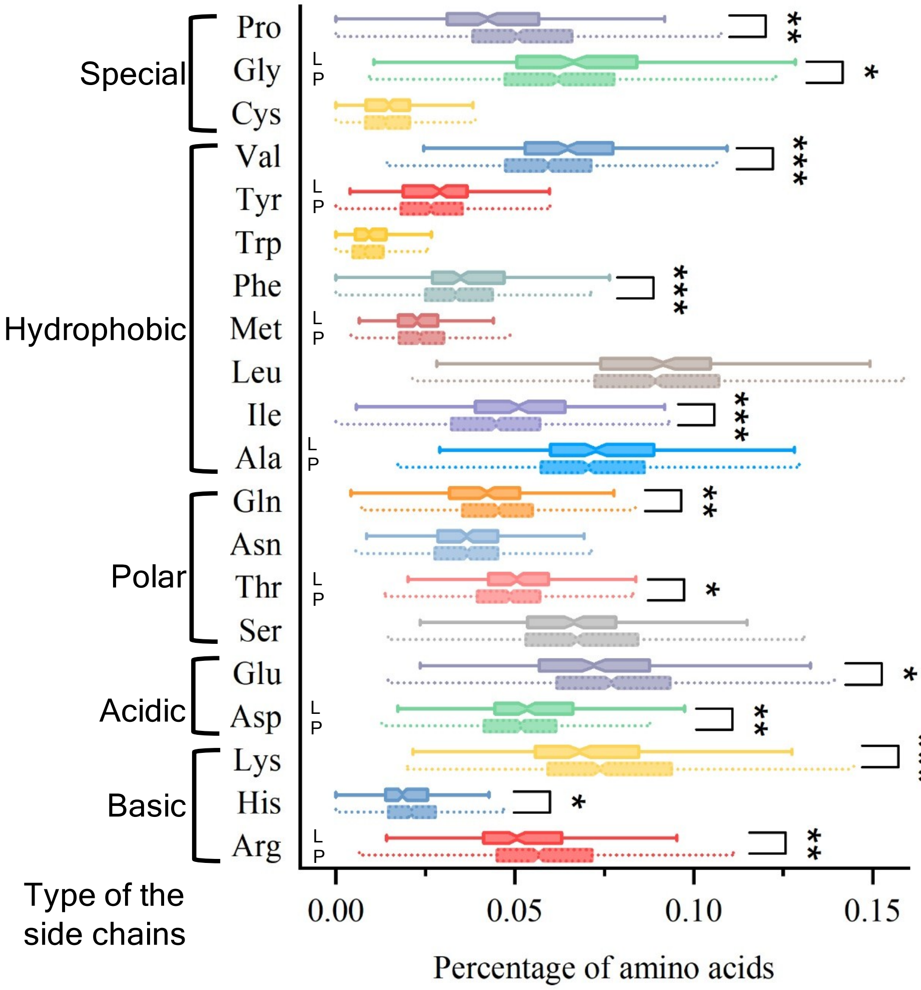

Figure S4. Percentage of amino acids in the proteins degraded through either the ubiquitin-proteasome pathway (P) or the autophagy-lysosome pathway (L) were assessed by the independent T-test (significance levels are marked as *** ($P < 0.001$), **($P < 0.01$) and *($P < 0.05$)).

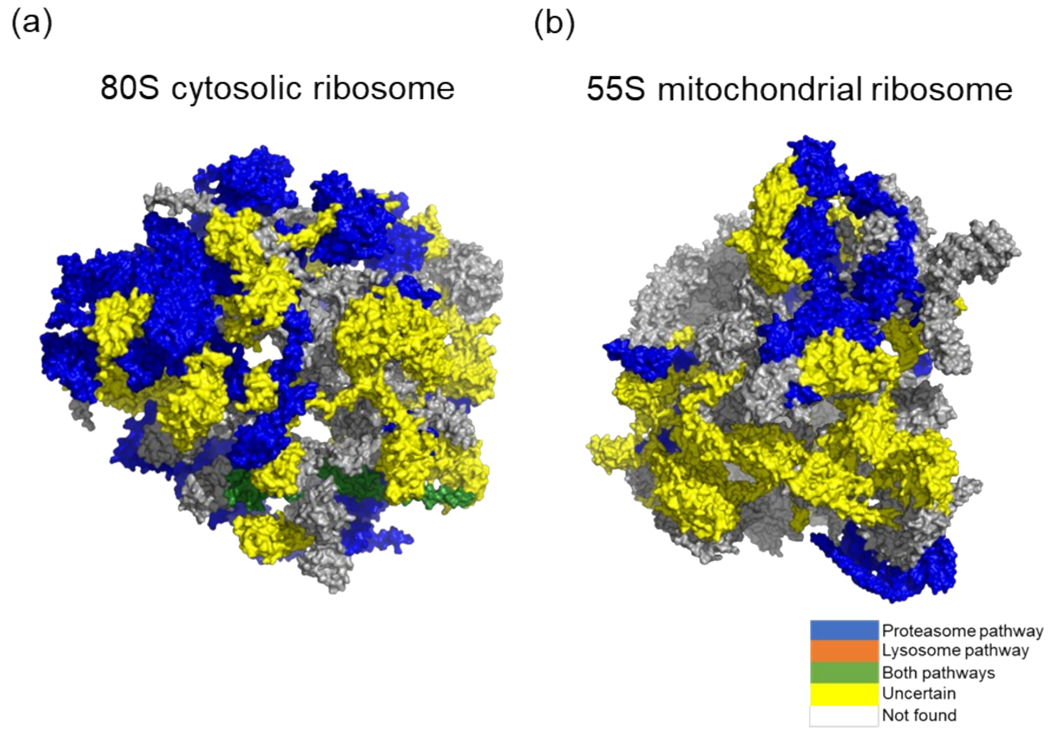

Figure S5. Degradation of proteins from the cytosolic ribosome and mitochondrial ribosome complexes. The degradation pathway of each protein is shown in the structure of the (a) cytosolic and (b) mitochondrial ribosomes. Each protein is colored based on its degradation pathway. Proteins degraded through the ubiquitin-proteasome pathway are in blue, those through the autophagy-lysosome pathway are in orange, those through both pathways are in green, proteins with an uncertain degradation pathway are in yellow, and unquantified proteins are in white.
